# Atypical Cadherin FAT3 Is a Novel Mediator for Morphological Changes of Microglia

**DOI:** 10.1523/ENEURO.0056-20.2020

**Published:** 2020-12-15

**Authors:** Tomomi Okajima, Yichen Gu, Rin-ichiro Teruya, Sarasa Yano, Takumi Taketomi, Ban Sato, Tomoki Chiba, Fuminori Tsuruta

**Affiliations:** 1 Faculty of Life and Environmental Sciences, University of Tsukuba, Tsukuba, Ibaraki 305-8577, Japan; 2 Graduate School of Life and Environmental Sciences, University of Tsukuba, Tsukuba, Ibaraki 305-8577, Japan; 3 College of Biological Sciences, School of Life and Environmental Sciences, University of Tsukuba, Tsukuba, Ibaraki 305-8577, Japan; 4 Ph.D. Program in Human Biology, School of Integrative and Global Majors, University of Tsukuba, Tsukuba, Ibaraki 305-8577, Japan; 5 Master's and Doctoral Program in Neuroscience, Graduate School of Comprehensive Human Sciences, University of Tsukuba, Tsukuba, Ibaraki 305-8577, Japan

**Keywords:** FAT3, HPRT1, hypoxanthine, Lesch–Nyhan syndrome, microglia, morphogenesis

## Abstract

Microglia are resident macrophages that are critical for brain development and homeostasis. Microglial morphology is dynamically changed during postnatal stages, leading to regulating synaptogenesis and synapse pruning. Moreover, it has been well known that the shape of microglia is also altered in response to the detritus of the apoptotic cells and pathogens such as bacteria and viruses. Although the morphologic changes are crucial for acquiring microglial functions, the exact mechanism which controls their morphology is not fully understood. Here, we report that the FAT atypical cadherin family protein, FAT3, regulates the morphology of microglial cell line, BV2. We found that the shape of BV2 becomes elongated in a high-nutrient medium. Using microarray analysis, we identified that FAT3 expression is induced by culturing with a high-nutrient medium. In addition, we found that purinergic analog, hypoxanthine, promotes FAT3 expression in BV2 and mouse primary microglia. FAT3 expression induced by hypoxanthine extends the time of sustaining the elongated forms in BV2. These data suggest that the hypoxanthine-FAT3 axis is a novel pathway associated with microglial morphology. Our data provide a possibility that FAT3 may control microglial transitions involved in their morphologic changes during the postnatal stages *in vivo*.

## Significance Statement

Microglia play an important role in regulating neural circuit formation and brain homeostasis. Microglial morphology is greatly changed from reactive form to surveillant form after birth. Intriguingly, these morphologic changes are tightly associated with regulating higher brain functions. However, the mechanisms of morphologic changes have not been elucidated. Here, we report that atypical cadherin family protein, FAT3, is a novel regulator for microglial morphology. We found that FAT3 expression is induced by purinergic derivative, hypoxanthine, and is necessary for sustaining microglial morphology after changing to elongated shapes *in vitro*. Because both purinergic metabolism and FAT3 pathway have been implicated in neurodevelopmental disorders, our findings may shed light on the novel approach for treating these disorders associated with a microglial abnormality.

## Introduction

Microglia play important roles in regulating not only the immune system but also neural circuit construction in the CNSs (Kettenmann et al., 2011). Previous study has reported that microglia exhibit the amoeboid shape during prenatal period and gradually transform into ramified shape around postnatal day (P)14. Usually, amoeboid microglia are thought to be reactive forms, which possess a high ability for engulfment. Because neuronal cell deaths frequently occur during the developmental stages, reactive microglia eliminate the apoptotic or vulnerable neurons to regulate the number of neurons and brain environments (Marín-Teva et al., 2004; Cunningham et al., 2013). On the other hand, microglia have been known to be involved in neural circuit formation through regulating neuronal survival, apoptosis, synaptogenesis, and synapse pruning (Marín-Teva et al., 2004; Sierra et al., 2010; Paolicelli et al., 2011; Cunningham et al., 2013; Ueno et al., 2013; Miyamoto et al., 2016). In particular, the control of synaptic connectivity is a key process that underlies the higher brain functions. Previous studies have shown that the number of synapses is dramatically increased during brain development, followed by reducing them by microglia (Huttenlocher and Dabholkar, 1997; Paolicelli et al., 2011; Schafer et al., 2012). In the case of normal development in mice, the synaptic pruning occurs approximately from P20 to P30. At this time, microglial morphology exhibits the ramified shape, which is a surveillance mode. Ramified microglia constantly elongates and retracts their processes to monitor the neural circuit even in the resting state (Nimmerjahn et al., 2005; Haynes et al., 2006). Finally, microglia contribute to the proper neural circuit formation by removing unnecessary synapses (Paolicelli et al., 2011; Schafer et al., 2012). It is thought that these morphologic alterations in microglia are tightly regulated not only by genetic programs but also by various factors, such as metabolites and exogenous factors, followed by constructing precise neural circuit and maintaining normal brain functions. Although the morphologic changes of microglia are implicated in their activity, the cues that modulate microglial morphology, particularly the intrinsic substances, have not been fully explicated.

The cadherin family proteins play an important role in brain development, such as synapse connectivity and neural circuit formation (Takeichi, 2007). In addition, mutations in cadherin family genes cause psychiatric and developmental disorders, including schizophrenia and autism spectrum disorder (ASD) (Redies et al., 2012). Thus, cadherin proteins in the brain are crucial for acquiring proper brain functions. So far, several groups have reported that atypical cadherins are expressed in the CNS and tightly regulate brain development. FAT3, which is one of the four FAT-related genes (*Fat1*-*Fat4*), is known to be abundantly expressed in the mammalian brain (Nagae et al., 2007; Zhang et al., 2014). *Fat3* gene encodes the transmembrane protein that possesses large extracellular domains and is an ortholog of the *Drosophila fat-like* gene. In *Drosophila*, Fat-like induces aberrant actin filament orientation in the ovary (Viktorinová et al., 2009). In the CNS, FAT3 is mostly expressed in neurons (Zhang et al., 2014, 2016) and regulates neuronal morphology in the retina (Deans et al., 2011; Krol et al., 2016). On the other hand, the functions of FAT3 in microglia have not been elucidated although FAT3 is expressed in microglia during the postnatal stage (Bennett et al., 2016).

Recent studies have reported that purinergic signaling plays an important role in regulating aspects of microglia under brain development (Fields and Burnstock, 2006). Purine derivatives, including ATP and ADP, are released from cells to the extracellular region and activate microglia via purinergic receptors, and thus it is crucial for various physiological processes in the CNS. Hypoxanthine, one of the purine derivatives, is implicated in brain development under the postnatal stage. For instance, newborns of mammals are exposed to hypoxic conditions at the time of birth, and hypoxanthine may become a marker of hypoxia. Indeed, abnormal hypoxic conditions trigger aberrant brain development (Saugstad, 1988). Moreover, it is known that perturbed hypoxanthine concentration is tightly associated with ASD (Gevi et al., 2016). Thus, precise regulation of purine derivatives, such as hypoxanthine, is essential for the normal brain development.

In this study, we found that the expression of atypical cadherin, FAT3, alters the morphology of microglia BV2 cells. Moreover, hypoxanthine induces FAT3 expression associated with their morphology. Our data provide the first evidence that links nucleic acid metabolism in microglia to their morphologic regulation.

## Materials and Methods

### Cell culture and stimulation

BV2 cells were cultured in either DMEM (high glucose; Wako) or DMEM/nutrient mixture F-12 (DMEM/F12; Thermo Fisher Scientific) containing 10% fetal bovine serum (FBS), 100 units penicillin and 100 mg streptomycin (P/S; Thermo Fisher Scientific). For screening, BV2 cells were cultured in DMEM with 10% FBS and P/S in the presence or absence of 150 μM L-alanine (Wako), 50 μM L-glutamic acid (Wako), 50 μM L-aspartic acid (Wako), 50 μM L-asparagine (Wako), 150 μM L-proline (Wako), 0.0052 pM CuSO_4_ (Wako), 1.5 pM FeSO_4_ (Wako), 1.5 pM ZnSO_4_ (Wako), 150 nM linoleic acid (Wako), 500 nM lipoic acid (Sigma), 15 nM biotin (Sigma), 500 nM vitamin B12 (Wako), and 15 μM hypoxanthine (Sigma). Pyridoxalphosphate-6-azophenyl-2',4'-disulfonic acid tetrasodium salt (PPADS) was purchased from Alexis Corporation. Dipyridamole (DY) and allopurinol (ALP) were purchased from Tokyo Chemical Industry. Hoechst 33342 was purchased from Thermo Fisher Scientific. DAPI was purchased from Dojindo. FAT3 siRNAs (NM_001080814, siRNA ID; SASI_Mm02_00293873) and MISSION siRNA universal negative control (SIC001) were purchased from Sigma; 50 nM FAT3 siRNAs were transfected using Lipofectamine RNAi Max Reagent (Thermo Fisher Scientific) according to the manufacturer’s instructions.

### Plasmid construction and antibody

A cDNA fragment encoding a C terminus (CT) of human-FAT3 containing 276 residues (4313–4589 amino acid) was amplified from FAT3 cDNA (product ID: ORK04991; Kazusa) and subcloned into the BamHI site of pET28(a) and the BglII site of pCS4-HA. The primers used were as follows:

FAT3_HA forward, 5′-AAGGATCCGCCACCCGCAAGAAGGTCTTCCGC-3′;

FAT3_HA reverse, 5′-CCGGATCCCTACACTTGAGTCTGATGCTGAGTCTCCAC-3′;

FAT3_recombinant forward, 5′-AAGGATCCGCCACCCGCAAGAAGGTCTTCAGAAAG-3′;

FAT3_recombinant reverse, 5′-AAGGATCCCTACACCTGGGTCTGCTGCTGTG-3′.

The His-tagged recombinant protein was produced in *Escherichia coli* BL21. The His-tagged FAT3 recombinant protein was purified using a Ni-Sepharose 6 Fast Flow (GE Healthcare), emulsified with Freund’s complete and incomplete adjuvant (Difco Laboratories), and was injected intradermally into female New Zealand white rabbits (Kitayama Labes). The FAT3 antibodies were purified by fractionation with ammonium sulfate (0–40% saturation), followed by immunoaffinity chromatography on a CNBr-activated Sepharose 4B (GE Healthcare) column that had been conjugated with GST-fused FAT3 CT protein carrying the C-terminal 276-residues sequence. FAT3 C-terminal antibody was concentrated 5× with a Vivaspin-100K (GE Healthcare). For immunoblot analyses, anti-HA (3F10, Roche) and anti-tubulin (DM1A, Sigma) antibodies were used as a primary antibody.

### RT-qPCR

BV-2 cells (1.5 × 10^5^ cells/60-mm dish) were cultured for 24 h at 37°C with 5% CO_2_ and treated with various stimulations for 24 h. Total RNAs from cells were isolated by ISOGEN II (NIPPON GENE) according to the manufacturer’s instructions. The cDNAs were synthesized by reverse transcriptase, 100 units ReverTra Ace (TOYOBO) together with 25 pmol Random Primer (nonamer; TOYOBO), 20 nmol dNTPs and 1.0 μg total RNAs. The qPCR was performed in triplicate in 96-well plate (Thermo Fisher Scientific) using THUNDERBIRD SYBR qPCR Mix (TOYOBO) in Thermal Cycler Dice Real Time System TP800 (TAKARA) or Applied Biosystems 7900HT Fast Real Time PCR System (Applied Biosystems). The relative quantity of the target expression was calculated by 2^-ΔΔCt^ methods using Thermal Cycler Dice Real Time System Software (TAKARA) or SDS Software 2.4.2 (Applied Biosystems) with the following calculation. The relative quantity = 2^−ΔΔCt^, ΔΔCt = (Ct^target^ – Ct^5S^) _sample_ – (Ct^target^ – Ct^5S^)_reference_; Ct, threshold cycle.

The primers used were as follows:

FAT3 forward, 5′-TATAGGAACGTGCGCTGCACCTGTAATG-3′;

FAT3 reverse, 5′-TCATAGCCCACACATTGCATGTCCTCTG-3′;

5S rRNA forward, 5′-CGGCCATACCACCCTGAAC-3′;

5S rRNA reverse, 5′-GCGGTCTCCCATCCAAGTAC-3′;

### Morphologic assay

BV2 cells were plated at the density of 5.0 × 10^4^ cells/35 mm dish under DMEM/F12 and incubated for 24 h at 37°C with 5% CO_2_. The DMEM/F12 media were replaced with either DMEM or DMEM/F12 in the presence or absence of each stimulation, and cells were incubated for 24 h. The images were collected using BIOREVO BZ-9000 (Keyence) equipped with 20× S. Plan Fluor 0.45 NA objective lenses (Nikon) controlled by BZ-II software (Keyence). Cell shapes were quantified by FIJI ImageJ software and calculated Feret diameters as a parameter of morphology (https://imagej.nih.gov/ij/docs/menus/analyze.html). To analyze the morphology, we took the 5–10 images (2.0 × 10^5^ μm^2^/field). The elongated cells were defined by a Feret diameter score higher than or equal to 2. The number of samples in each experiment is given in Table 1.

**Table 1:** The number of cells counted for the morphology assay

Figure 1*B*											
Field number	1	2	3	4	5						
DMEM	132	107	120	102	111						
DMEM/F12	78	98	121	102	84	(cells)					
Figure 1*L*											
Field number	1	2	3	4	5	6	7	8	9	10	
DMEM+siControl	65	67	59	69	83	32	35	50	47	47	
DMEM/F12+siControl	71	53	73	60	73	96	66	65	92	72	
DMEM/F12+siFat3	63	57	53	76	56	60	89	81	49	102	(cells)
Figure 2*F*											
Field number	1	2	3	4	5						
DMEM	109	109	81	134	99						
DMEM/F12	155	136	127	131	129						
DMEM+HYP	91	131	75	68	78	(cells)					
Figure 3*C*											
Field number	1	2	3	4	5						
DMEM	72	103	102	87	118						
DMEM/F12	74	109	108	130	124						
DMEM+HYP	57	57	87	59	87						
DMEM+HYP+PPADS	113	85	78	71	107	(cells)					
Figure 3*H*											
Field number	1	2	3	4	5						
DMEM	79	92	110	117	108						
DMEM+DY	105	157	146	173	103	(cells)					
Figure 3*K*											
Field number	1	2	3	4	5						
DMEM	120	79	111	94	120						
DMEM+ALP	138	168	115	104	104	(cells)					
Figure 4*F*											
Field number	1	2	3	4	5						
DMEM (conditioned)	72	64	57	103	52						
DMEM/F12 (conditioned)	43	39	123	87	63						
DMEM+HYP (conditioned)	61	72	91	77	89						
DMEM (fresh)	101	109	79	88	85						
DMEM/F12 (fresh)	100	124	84	87	97						
DMEM+HYP (fresh)	88	110	88	78	50	(cells)					

### Immunoblot analysis

HEK293T cells were plated at 5.0 × 10^5^ cells on six-well plate and incubated at 37°C with 5% CO_2_ for 1 d. Cells were transfected using 1.0 μg/μl polyethyleneimine MAX (Polyscience) and collected with lysis buffer [20 mM Tris-HCl (pH 8.0), 150 mM NaCl, 1 mM EDTA, 0.5% NP-40, and 1 mM DTT]. Cell lysates were centrifuged at 14,000 rpm for 5 min. The supernatant was run on SDS-PAGE for protein separation, followed by electrophoretic transfer to a polyvinylidene difluoride membrane (Pall). After 1 h blocking by 5% skim milk at room temperature, membranes were incubated with primary antibodies overnight at 4°C. The proteins on membrane were then detected with HRP-conjugated secondary antibodies and chemiluminescence reagents [ECL Prime Western Blotting Detection Reagents (GE Healthcare) or Chemi-Lumi One Super (Nacarai tesque)].

### Immunocytochemistry

BV2 cells were plated at 5.0 × 10^4^ cells and incubated on 15 mm coverslips in 12-well plates at 37°C with 5% CO_2_ in each condition. The cells were fixed with 4% paraformaldehyde (Merck KGaA) in PBS for 10 min at room temperature. The coverslips were washed in PBS and blocked with 5% bovine serum albumin (BSA; Wako) in PBS with 0.4% Triton X-100 (MP Biomedicals), then incubated with the FAT3 antibody (1:250) for overnight at 4°C. After a PBS wash, samples were incubated with the secondary antibody; Alexa Fluor 488 anti-rabbit IgG (1:500; Thermo Fisher Scientific) for 30 min at room temperature in blocking solution. Cells were imaged with a confocal laser scanning fluorescence microscopy (LSM700; Carl Zeiss) and BIOREVO BZ-9000 (Keyence).

### Microarray analysis

BV2 cells were plated at 3.0 × 10^5^ cells on a 60 mm dish in the DMEM/F12, and culture media were exchanged with either DMEM or DMEM/F12 on the next day. After 1 d of incubation, total RNAs were extracted using ISOGEN II (NIPPON GENE) according to the manufacturer’s instructions. The single-stranded cDNAs were generated from total RNA (100 ng) using Ambion WT Expression kit (Ambion Inc), sequentially fragmented and covalently linked to biotin using GeneChip WT Terminal Labeling and Hybridization kit (Affymetrix) according to the manufacturer’s instructions. These samples were incubated at 45°C at 60 rpm for 17 h to hybridize on Affymetrix GeneChip Mouse Gene 1.0 ST arrays. After hybridization, each probe array was washed and stained with Affymetrix GeneChip Fluidics Station 450, and scanned by Affymetrix GeneChip Scanner 3000. Data were analyzed with Affymetrix Expression Console software and Affymetrix Transcriptome Analysis Console. The GEO accession numbers for the array dataset are GSM4306479 and GSM4306480.

### Measurement of intracellular hypoxanthine concentration

BV2 cells were incubated at 1.0 × 10^6^ cells on a 100-mm dish in the DMEM together with 15 μM hypoxanthine for 12 h, sequentially treated with 10 μM DY or 10 μM ALP for 3 h. BV2 cells were incubated with ice-cold xanthine assay buffer (BioVison) for 10 min on ice and subjected to measurement of intracellular hypoxanthine concentration according to the manufacturer’s instructions of the xanthine/hypoxanthine colorimetric/fluorometric assay kit (BioVision).

### Live cell imaging analysis

BV2 cells were plated at 1.0 × 10^5^ cells in a 35 mm dish and incubated for 24 h. Cells were replaced with DMEM containing 1.0 ng/μl LPS in the presence or absence of 15 μM hypoxanthine and were then subjected to the imaging analysis. Live cell imaging was conducted on BIOREVO BZ-9000 (Keyence) equipped with 20× S. Plan Fluor 0.45 NA objective lenses (Nikon) controlled by BZ-II software (Keyence). BV2 cells were imaged at one frame per 15 min for 12 h. The images were analyzed using FIJI ImageJ software. Briefly, the Feret diameter in each cell was quantified using the function of Set Measurements in Fiji ImageJ. The elongated cells were defined by a Feret diameter score ≥3.

### Primary culture microglia

All animal experiments were conducted according to the guidelines for animal care and use. The monoclonal CD11b antibody is generated using the M1/70 hybridoma cell line (M1/70.15.11.5.2-f, Developmental Studies Hybridoma Bank, The University of Iowa) grown in Hybridoma-SFM (Thermo Fisher Scientific). The medium containing the CD11b antibody was collected by centrifugation when cells were grown until confluent. One day before dissection of the mouse, the sterile 100-mm Petri dishes were coated by 6 mg/ml goat-anti-mouse IgG secondary antibody (Jackson ImmunoResearch) in a solution of 50 mM Tris-HCl (pH 9.5) for 1 h at 37°C, followed by washing with PBS three times. The CD11b antibodies were added to the coated dishes and incubated overnight. ICR pups (P1–P3; SLC) were rapidly dissected to take out the brains. These brains were placed into ice-cold PBS and chopped with a scalpel blade and surgical scissors. The chopped brains were suspended in 5 ml of ice-cold PBS containing 40 units DNaseI (Wako) and were transferred to a Dounce homogenizer equipped with a loose pestle (DWK Life Sciences GmbH). They were homogenized 10 times and were then centrifuged at 500 × *g* for 15 min at 4°C. After discarding the supernatant, cell pellets were resuspended in 5 ml of PBS containing 2 mg/ml peptone from milk solids (Sigma), and passed through a 70-μm cell strainer (BD Biosciences). To conduct a positive selection, the cells were incubated on immunopanning dishes coated with the CD11b antibodies for 20 min at room temperature. Unattached cells and debris were removed by washing using PBS without Ca^2+^ and Mg^2+^ 10 times. The attached cells on the immunopanning dishes were treated with 0.25% trypsin/1 mM EDTA for 1 min at 37°C. After incubation, the trypsin was quenched by 4 ml of DMEM with 10% FBS. The cells were centrifuged at 500 × *g* for 15 min at 4°C and resuspended with 1 ml of DMEM with 10% FBS. For immunocytochemistry, the coverslips were coated with Bovine Collagen Coating Solution (CELL Applications) for 15 min at 37°C. After aspirating the collagen spots, the suspended cells were immediately plated at 3.5 × 10^3^ cells/spot and incubated for 10 min at 37°C. The attached cells were incubated under 500 μl of either DMEM, DMEM/F12, or DMEM with 15 μM hypoxanthine for 24 h at 37°C with 5% CO_2_. For RNA isolation, the 24-well plate was coated with Bovine Collagen Coating Solution for 1 h at 37°C. The cells were plated at 3 × 10^4^ cells and incubated in either 500 μl of DMEM, DMEM/F12, or DMEM treated with 15 μM hypoxanthine for 24 h at 37°C with 5% CO_2_.

### Statistical analysis

All experiments were performed in at least two independent experiments. All statistical data were analyzed by using GraphPad Prism (GraphPad Software) compared by Student’s *t* test and one-way ANOVA. The statistical analyses of qRT-PCR are shown in Table 2.

**Table 2:** Statistical analysis for the qPCR experiments

BV-2 qPCR (Fig. 1*F*)							
Unpaired *t* test	Mean difference	95.00% CI of difference	Significant?	Summary	*p* value		
DMEM vs DMEM/F12	–4.973 ± 0.4619	–6.256 to –3.691	Yes	***	0.0004		
BV-2 siRNA qPCR (Fig. 1*L*)							
Tukey's multiple comparisons test	Mean difference	95.00% CI of difference	Significant?	Summary	Adjusted *p* value		
DMEM vs DMEM/F12	3.192	–0.3346 to 6.718	No	ns	0.0719	A-B	
DMEM vs siFat3	–2.381	–5.907 to 1.146	No	ns	0.1763	A-C	
DMEM/F12 vs siFat3	–5.572	–9.099 to –2.046	Yes	**	0.0068	B-C	
BV-2 NEAAs metals others qPCR (Fig. 2*B*)							
Dunnett's multiple comparisons test	Mean difference	95.00% CI of difference	Significant?	Summary	Adjusted *p* value	A-?	
DMEM vs DMEM/F12	2.113	1.032 to 3.195	Yes	***	0.0007	B	DMEM/F12
DMEM vs NEAAs	0.5533	–0.5281 to 1.635	No	ns	0.4259	C	NEAAs
DMEM vs metals	0.4133	–0.6681 to 1.495	No	ns	0.6518	D	Metals
DMEM vs others	2.113	1.032 to 3.195	Yes	***	0.0007	E	Others
BV-2 others qPCR (Fig. 2*C*)							
Dunnett's multiple comparisons test	Mean difference	95.00% CI of difference	Significant?	Summary	Adjusted *p* value	C-?	
DMEM vs DMEM/F12	2.858	1.779 to 3.936	Yes	****	<0.0001	A	DMEM/F12
DMEM vs HYP	6.176	5.098 to 7.255	Yes	****	<0.0001	B	HYP
DMEM vs biotin	–0.5829	–1.661 to 0.4955	No	ns	0.4517	D	Biotin
DMEM vs linoleic	1.584	0.5057 to 2.663	Yes	**	0.0038	E	Linoleic
DMEM vs VB12	–0.9733	–2.052 to 0.1052	No	ns	0.0839	F	VB12
DMEM vs lipoic	–0.6831	–1.761 to 0.3954	No	ns	0.3094	G	Lipoic
Primary microglia qPCR (Figure 2*I*)							
Dunnett's multiple comparisons test	Mean difference	95.00% CI of difference	Significant?	Summary	Adjusted *p* value	A-?	
DMEM vs DMEM/F12	1.35	0.629 to 2.06	Yes	**	0.003	B	DMEM/F12
DMEM vs HYP	1.15	0.436 to 1.87	Yes	**	0.007	C	HYP

## Results

### FAT3 regulates BV2 morphology

**Figure 1.**
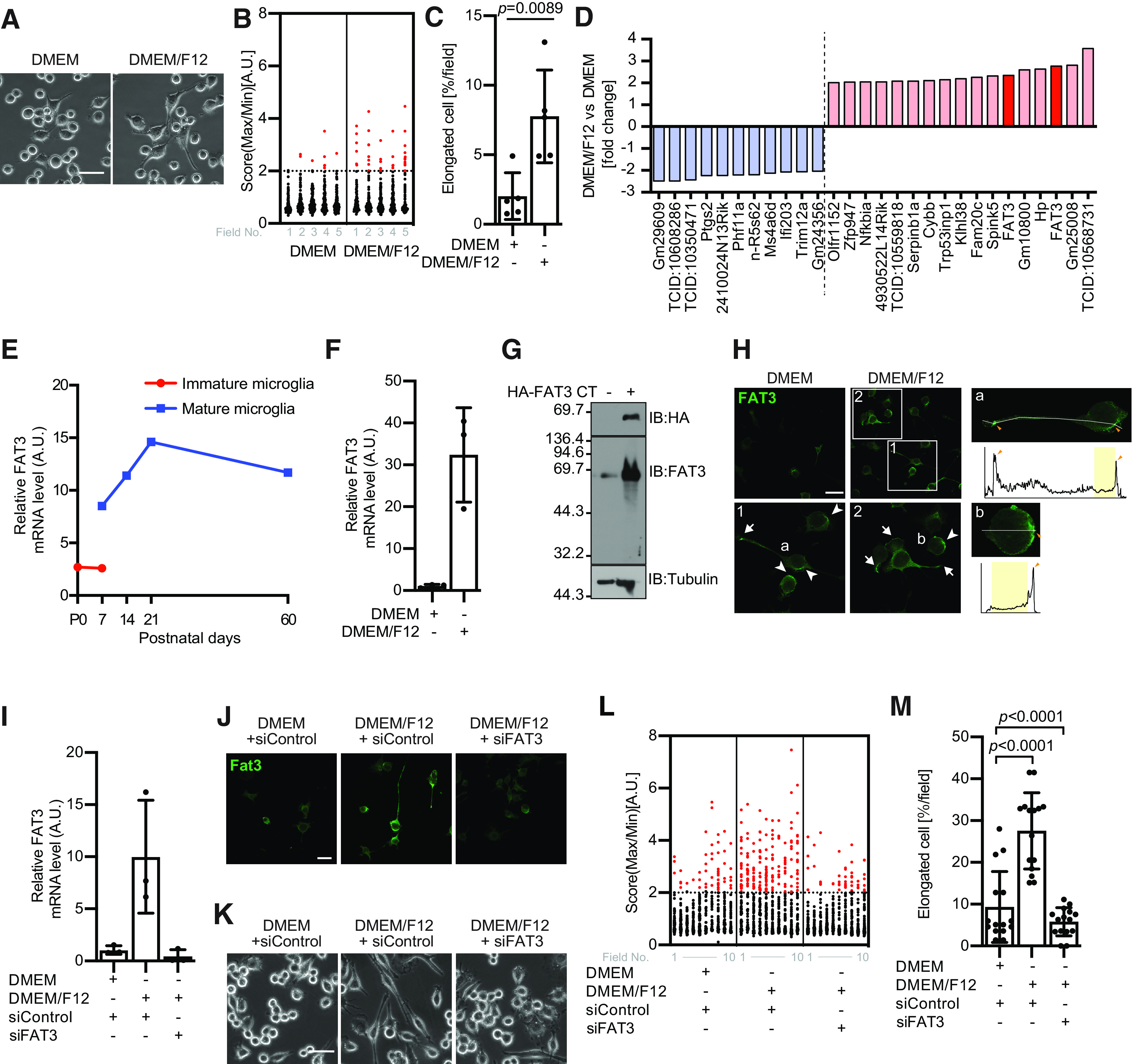
FAT3 regulates BV2 morphology. ***A***, BV2 cells were incubated with either DMEM as control medium or high-nutrient medium, DMEM/F12 for 24 h. Scale bar: 50 μm. ***B***, Quantification of data from ***A***. The score indicates a ratio of maximum to minimum Feret diameter; *n* > 78 cells; 5 fields. The elongated cell is defined as the score over 2. ***C***, The graph shows the relative percentage of the elongated cells in each field; *n* = 5 fields; mean ± SD; *p* value was calculated by Student’s *t* test. The data were reproduced in at least five independent experiments. ***D***, A subset of mRNAs identified in microarray experiments. The graph indicates the top 17 of most upregulated and 11 of most downregulated genes under DMEM/F12 culture condition. ***E***, The graph indicates *in silico* analysis of microglial FAT3 expression level from P0 to P60 in mice. The values were obtained from the Brain RNA-Seq database (http://www.brainrnaseq.org/). The red line indicates immature microglia (Tmem119^–^ cells), and the blue line indicates mature microglia (Tmem119^+^ cells). ***F***, BV2 cells were incubated with either DMEM/F12 or DMEM for 24 h. FAT3 mRNAs were measured by quantitative RT-PCR analysis and normalized to 5S ribosomal RNA; *n* = 3; mean ± SD. The data were reproduced in at least four independent experiments. ***G***, HEK293T cells were transfected with HA-FAT3 CT (4313-4589) and subjected to immunoblot analysis with anti-FAT3, anti-HA, and anti-tubulin antibodies. ***H***, left, BV2 cells were incubated with either DMEM/F12 or DMEM for 24 h. Immunostaining showing the localization of FAT3 cultured in either DMEM or DMEM/F12. Arrow, FAT3 expression in the distal processes and the edge of lamellipodia. Arrowhead, FAT3 in the proximal region. Scale bar: 50 μm. Right, The line profiles of FAT3 staining. The yellow areas indicate the nuclei regions. The orange arrowheads indicate accumulated FAT3 regions. ***I***, BV2 cells were incubated for 24 h after transfection of FAT3 siRNA. FAT3 mRNAs were measured by quantitative RT-PCR analysis and normalized to 5S ribosomal RNA; *n* = 3; mean ± SD. The data were reproduced in at least three independent experiments. ***J***, Immunostaining showing the FAT3 localization in the presence or absence of FAT3 siRNA for 24 h. Scale bar: 50 μm. ***K***, BV2 cells were incubated in the presence or absence of FAT3 siRNA for 24 h. Scale bar: 50 μm. ***L***, Quantification of data from ***K***. The score indicates a ratio of maximum to minimum Feret diameter; *n* > 32 cells; 10 fields. The elongated cell is defined as the score over 2. ***M***, The graph shows the relative percentage of the elongated cells in each field; *n* = 10 fields; mean ± SD; *p* value was calculated by one-way ANOVA. The data were reproduced in at least three independent experiments.

To understand the mechanisms of how microglial morphology is controlled, we cultured microglial cell line BV2 with either a standard medium, DMEM, or a high-nutrient medium, DMEM/F12. When BV2 were cultured in DMEM, most cells exhibited round shapes. On the other hand, when cells were cultured in DMEM/F12, the number of elongated cells was increased (Fig. 1*A–C*), indicating that microglial morphology is influenced by the surrounding culture condition.

Next, to investigate the specific genes regulating BV2 morphology under DMEM/F12 conditions, we conducted a microarray analysis. The global gene expression patterns were different for each condition. Some genes were significantly upregulated under DMEM/F12 (Fig. 1*D*; Extended Data Table 1-1). Although we found several interesting targets at least in this screening, one of the most interesting candidates is *Fat3* gene. Because FAT3 has been reported to regulate neuronal morphology in retinal amacrine cells (Deans et al., 2011; Krol et al., 2016), we presumed that FAT3 also controls the microglial morphology. To confirm this idea, we surveyed the expression level of FAT3 in microglia using the Brain RNA-seq database (http://www.brainrnaseq.org/). Interestingly, FAT3 expression is augmented in correlation with the timing of microglial transition around P14 (Fig. 1*E*), suggesting that FAT3 is a potential candidate, which controls microglial morphogenesis. Then, we confirmed whether FAT3 expression in BV2 cells is affected by culture condition. FAT3 expression was upregulated when BV2 cells were cultured in DMEM/F12 (Fig. 1*F*). Particularly, it seems likely that DMEM/F12 facilitates FAT3 clustering in a distal process and a leading edge of lamellipodia. Also, FAT3 showed polarized localization in the cell body (Fig. 1*G*,*H*), demonstrating that DMEM/F12 regulates FAT3 expression locally. We next investigated whether FAT3 is necessary for regulating their morphology. To do this, we first checked the efficiency of FAT3 siRNA. Treatment with siRNA significantly reduced DMEM/F12-induced expression levels of mRNA and changed immunostaining patterns in BV2 cells (Fig. 1*I*,*J*). Using this siRNA, we examined the necessity of FAT3 for regulating BV2 morphology. We found that FAT3 siRNA significantly suppressed the morphologic changes even in DMEM/F12 culture conditions (Fig. 1*K–M*), suggesting that FAT3 regulates BV2 morphology depending on the culture condition.

10.1523/ENEURO.0056-20.2020.t1-1 Table 1-1. Relative gene expression in BV2 cells (DMEM/F12 vs DMEM). Download Table 1-1, XLSX file.

### Hypoxanthine promotes FAT3 expression in microglia

**Figure 2.**
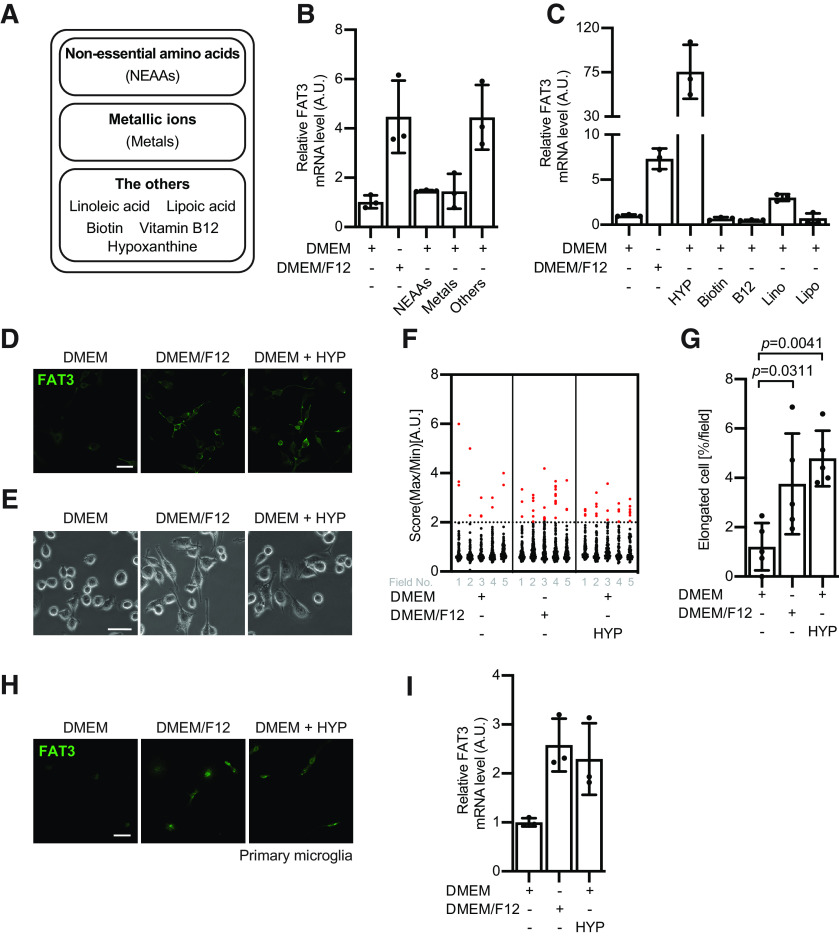
Hypoxanthine promotes FAT3 expression in microglia. ***A***, The chart summarizes the additional components of DMEM/F12 against DMEM. They are categorized into three large groups, non-essential amino acids (NEAAs), metallic ions (metals), and the others. ***B***, BV2 cells were incubated with the indicated components for 24 h. FAT3 mRNAs were measured by quantitative RT-PCR analysis and normalized to 5S ribosomal RNA; *n* = 3; mean ± SD. The data were reproduced in at least three independent experiments. ***C***, BV2 cells were incubated with 15 μM hypoxanthine (HYP), 0.5 μM vitamin B12 (B12), 15 nM biotin, 0.15 μM linoleic acid (Lino), or 0.5 μM lipoic acid (Lipo) for 24 h. The FAT3 mRNAs were measured by quantitative RT-PCR analysis and were normalized to 5S ribosomal RNA; *n* = 3; mean ± SD. The data were reproduced in at least four independent experiments. ***D***, BV2 cells were incubated with either DMEM, DMEM/F12, or DMEM with 15 μM hypoxanthine for 24 h. Immunostaining showing the FAT3 localization in the presence or absence of 15 μM hypoxanthine. Scale bar: 50 μm. ***E***, Phase contrast images showing BV2 morphology. Scale bar: 50 μm. ***F***, Quantification of data from ***E***. The score indicates a ratio of maximum to minimum Feret diameter; *n* > 68 cells; 5 fields. The elongated cell is defined as the score over 2. ***G***, The graph shows the relative percentage of the elongated cells in each field; *n* = 5 fields; mean ± SD; *p* value was calculated by one-way ANOVA. The data were reproduced in at least four independent experiments. ***H***, The primary microglia were incubated with either DMEM, DMEM/F12, or DMEM with 15 μM hypoxanthine for 24 h. Immunostaining showing the FAT3 expression in the presence or absence of 15 μM hypoxanthine. Scale bar: 50 μm. ***I***, The primary microglia were incubated with either DMEM, DMEM/F12, or DMEM with 15 μM hypoxanthine for 24 h. FAT3 mRNAs were measured by quantitative RT-PCR analysis and normalized to 5S ribosomal RNA; *n* = 3; mean ± SD. The data were reproduced in two independent experiments.

We next investigated which factors in DMEM/F12 are implicated in FAT3 expression in BV2 cells. To examine this, we divided the specific components of DMEM/F12, which are not included in DMEM, into three groups (i.e., non-essential amino acids, metallic ions, the others; Fig. 2*A*), treated BV2 with them, and quantified the amount of FAT3 mRNA expression. When BV2 were cultured with DMEM/F12, FAT3 expression was clearly enhanced. However, DMEM containing either “non-essential amino acids” or “metallic ions” has little effect on the expression of FAT3. On the other hand, treatment of BV2 cells with “the others” enhanced FAT3 expression (Fig. 2*B*), indicating that the specific factor contained in “the others” is required for the FAT3 expression. Next, we surveyed which factors in “the others” regulate BV2 morphology. To answer this question, we additionally divided “the others” into five factors (i.e., hypoxanthine, biotin, vitamin B12, linoleic acid, and lipoic acid) and treated the cells with these compounds individually. Accordingly, treatment with hypoxanthine clearly induced FAT3 expression; however, other factors such as biotin, vitamin B12, linoleic acid, and lipoic acid did not have significant influences on the induction of FAT3 expression (Fig. 2*C*). Moreover, hypoxanthine stimulation enhances the expression of FAT3 as well as DMEM/F12 culture conditions (Fig. 2*D*). Subsequently, to examine whether hypoxanthine is sufficient for the morphologic changes of BV2, we quantified the elongated shape of BV2 cells in the presence or absence of hypoxanthine. BV2 morphology exhibited an elongated shape in the DMEM containing hypoxanthine (Fig. 2*E–G*). To further confirm whether hypoxanthine enhances FAT3 expression in microglia, we purified the primary microglia from neonatal mice brains. Treatment with hypoxanthine increased FAT3 expression in primary microglia, as well as BV2 cells (Fig. 2*H*,*I*). These data suggest that hypoxanthine is a key mediator that induces FAT3 expression in microglia.

### Intracellular hypoxanthine is crucial for BV2 morphologic changes

**Figure 3.**
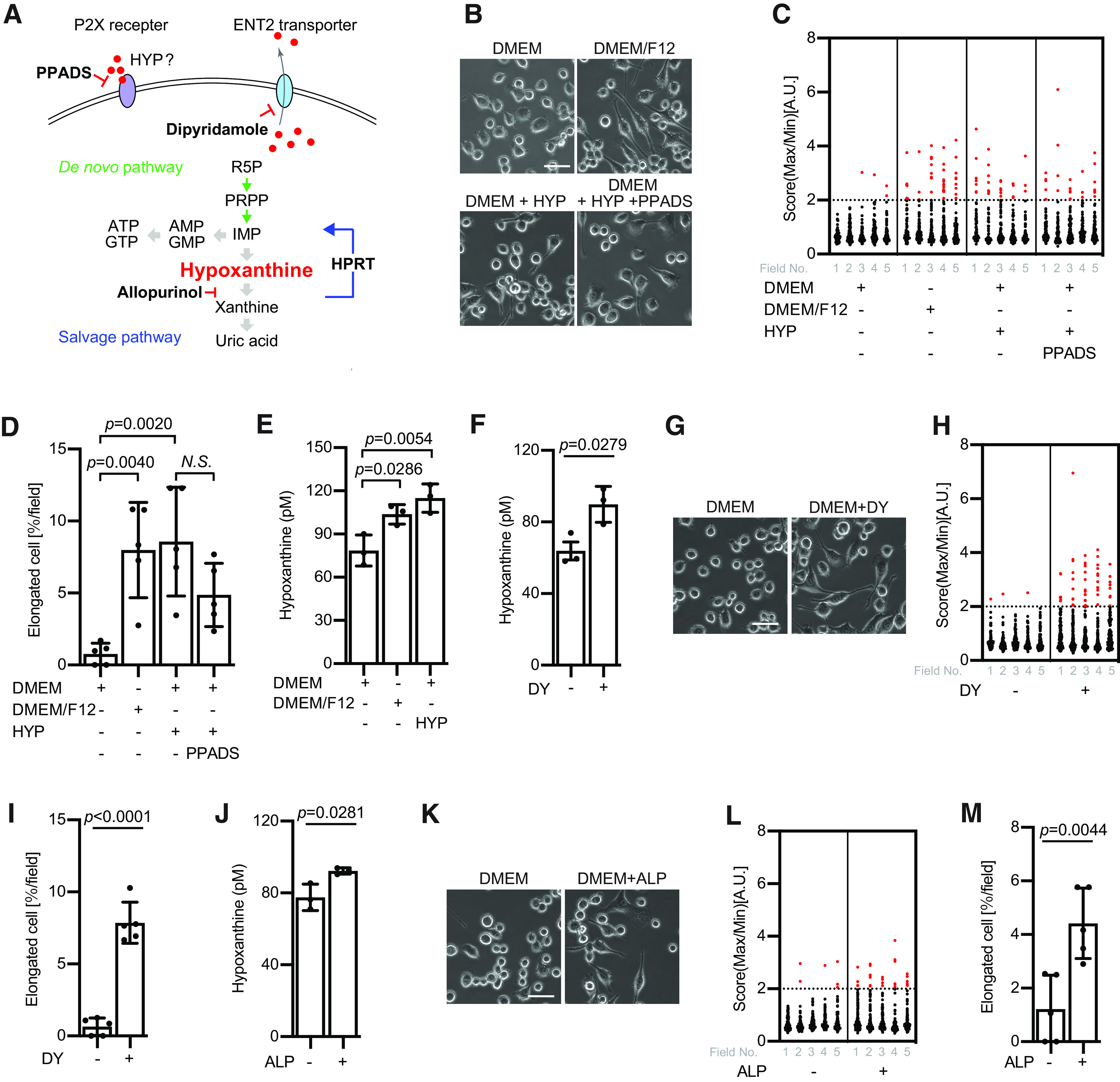
Intracellular hypoxanthine is crucial for BV2 morphologic changes. ***A***, Schematic overview of purinergic metabolisms. R5P; ribose 5-phosphate; PRPP, phosphoribosyl pyrophosphate. ***B***, BV2 cells were incubated in the presence or absence of 15 μM hypoxanthine (HYP) and 10 μM PPADS for 24 h. Scale bar: 50 μm. The data were reproduced in at least three independent experiments. ***C***, Quantification of data from ***B***. The score indicates a ratio of maximum to minimum Feret diameter; *n* > 57 cells; 5 fields. The elongated cell is defined as the score over 2. ***D***, The graph indicates the relative percentage of elongated BV2 cells in ***C***; *n* = 5 fields; mean ± SD; *p* value was calculated by one-way ANOVA. N.S., not significant. The data were reproduced in at least two independent experiments. ***E***, The graph indicates the intracellular hypoxanthine concentration cultured in DMEM, DMEM/F12, or DMEM with 15 μM hypoxanthine for 3 h; *n* = 3; mean ± SD; *p* value was calculated by one-way ANOVA. The data were reproduced in at least two independent experiments**. *F***, The graph indicates the intracellular hypoxanthine concentration in BV2 cells cultured in DMEM in the presence or absence of 10 μM DY for 3 h. Mean ± SD; *p* value was calculated by Student’s *t* test. ***G***, BV2 cells were incubated in the presence or absence of 10 μM DY cultured in DMEM for 24 h. Scale bar: 50 μm. ***H***, Quantification of data from ***G***. The score indicates a ratio of maximum to minimum Feret diameter; *n* > 79 cells; 5 fields. The threshold of the elongated cells was defined by setting over 2. ***I***, The graph indicates the relative percentage of elongated BV2 cells with or without DY in ***H***; *n* = 5 fields; mean ± SD; *p* value was calculated by Student’s *t* test. The data were reproduced in at least three independent experiments. ***J***, The graph indicates the intracellular hypoxanthine concentration in the presence or absence of 10 μM ALP cultured in DMEM for 3 h; *n* = 5; mean ± SD; *p* value was calculated by Student’s *t* test. The data were reproduced in at least two independent experiments. ***K***, BV2 cells were incubated in the presence or absence of 10 μM ALP cultured in DMEM for 24 h. Scale bar: 50 μm. ***L***, Quantification of data from ***K***. The score indicates a ratio of maximum to minimum Feret diameter; *n* > 79 cells; 5 fields. The elongated cell is defined as the score over 2. ***M***, The graph indicates the relative percentage of elongated BV2 cells in ***L***; *n* = 5 fields; mean ± SD; *p* value was calculated by Student’s *t* test. The data were reproduced in at least three independent experiments.

It has been known that P2X purinergic receptors play an important role in microglial functions. Then, we investigated whether the P2X purinergic receptor is a target of extracellular hypoxanthine using P2X purinergic receptor antagonist, PPADS (Fig. 3*A*). Consequently, PPADS did not significantly block hypoxanthine-induced morphologic changes of BV2 (Fig. 3*B–D*), implying that the P2X purinergic receptors have little effect on BV2 morphologic changes depending on extracellular hypoxanthine. Because incubation with either DMEM/F12 or hypoxanthine slightly increased a concentration of intracellular hypoxanthine (Fig. 3*E*), we investigated whether intracellular hypoxanthine regulates microglial morphology. It has been reported that treatment with an equilibrative nucleoside transporter (ENT) inhibitor, dipyridamole (DY), is capable of increasing intracellular hypoxanthine via blocking export hypoxanthine (Robillard et al., 2008; Fig. 3*A*). We confirmed whether DY increases a concentration of intracellular hypoxanthine in BV2 cells. We find that DY application slightly but significantly increased the hypoxanthine in BV2 cells (Fig. 3*F*). Using this condition, we quantified the BV2 morphology. The elongated BV2 cells were increased after the treatment with DY (Fig. 3*G–I*), implying that the intracellular concentration of hypoxanthine has an influence on BV2 morphology. To further confirm this, we used another inhibitor, allopurinol (ALP), which is a xanthine oxidase inhibitor (Fig. 3*A*). Since the xanthine oxidase is known to catalyze hypoxanthine to xanthine, inhibiting this enzyme also accumulates hypoxanthine in cells (Fig. 3*J*). As we expected, treatment with ALP increased the elongated shape of BV2 cells (Fig. 3*K–M*). Taken together, these data suggest that upregulations of intracellular hypoxanthine promote morphologic changes of BV2 cells.

### Hypoxanthine-FAT3 pathway controls the BV2 morphologic dynamics in concert with a humoral factor

**Figure 4.**
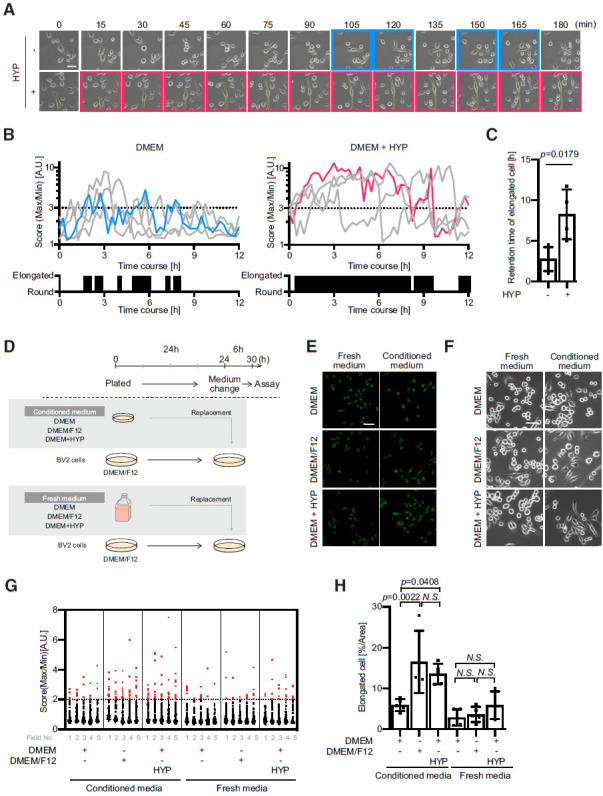
Hypoxanthine-FAT3 pathway controls the BV2 morphologic dynamics in concert with a humoral factor. ***A***, BV2 cells were imaged at one frame per 15 min over for 12 h with 1.0 μg/μl LPS in the presence or absence of 15 μM hypoxanthine (HYP). Representative images are shown. The blue and red lines indicate the elongated states. Scale bar: 20 μm. ***B***, The graph indicates the ratio of maximum to minimum Feret diameter. ***C***, The graph is summarized by data in ***B***; *n* = 4; mean ± SD; *p* values were calculated by Student’s *t* test. The data were reproduced in at least three independent experiments. ***D***, Scheme of experiment. BV2 cells were incubated with DMEM/F12 medium for 24 h and were then replaced with either conditioned or fresh media for 24 h. For the conditioned media, other BV2 cells were incubated for 24 h using indicated media. ***E***, BV2 cells were incubated for 6 h after exchanging media and were then subjected to immunocytochemistry. Immunostaining showing the expression level of FAT3. It seems that FAT3 expression is maintained after changing the media. Scale bar: 50 μm. ***F***, Phase contrast images showing BV2 morphology. Scale bar: 50 μm. ***G***, The score indicates a ratio of maximum to minimum Feret diameter; *n* > 39 cells; 5 fields. The elongated cell is defined as the score over 2. ***H***, The graph indicates the relative percentage of elongated BV2 cells morphology in ***F***; *n* = 5 fields; mean ± SD; *p* values were calculated by one-way ANOVA. N.S., not significant. The data were reproduced in at least two independent experiments.

Microglial morphology is highly dynamic even in the resting state (Nimmerjahn et al., 2005). Since FAT3 is expressed at the timing of microglial transition (Bennett et al., 2016), we expected that our findings provide potential clues that underlie their morphologic changes. Then, we investigated the mechanisms by which BV2 shapes were changed over time using live-cell imaging analyses (Fig. 4*A–C*; Movies 1, 2). The morphologic changes of BV2 were highly dynamic, and BV2 constantly shuttle between round and elongated shapes. Interestingly, treatment with hypoxanthine slightly but significantly prolonged the retention time of elongation forms, suggesting that FAT3 sustains elongated shapes and inhibits their retraction.

Movie 1. The time-lapse recoding of BV2 cells in the absence of hypoxanthine in Fig. 4*A*. 10.1523/ENEURO.0056-20.2020.video.1

Movie 2. The time-lapse recoding of BV2 cells in the presence of hypoxanthine in Fig. 4*A*. 10.1523/ENEURO.0056-20.2020.video.2

We next investigated the mechanisms of how BV2 shape is stabilized in the presence of hypoxanthine. Since a number of elongated BV2 were observed in low density cultured with DMEM/F12, we speculate that BV2 morphology is regulated by not only a cell-cell interaction but also a humoral factor, which is a potential ligand for FAT3. To answer this question, we cultured BV2 cells with or without the replacements of the medium. Consistent with our findings in Figure 1, both hypoxanthine and DMEM/F12 promoted the morphologic changes. On the other hand, the number of the round shape of BV2 was increased even in the DMEM/F12 condition and DMEM containing hypoxanthine when the medium was exchanged frequently (Fig. 4*D–H*). At least in this condition, it seems that an adequate amount of FAT3 was expressed when cells were incubated with DMEM/F12 for 24 h. Therefore, hypoxanthine-induced FAT3 expression is not sufficient for altering BV2 morphology further, implying that BV2 morphologic changes are mediated by the extracellular factors as well. Probably, both a secreted humoral factor and hypoxanthine-induced FAT3 are necessary for regulating BV2 morphogenesis.

## Discussion

**Figure 5.**
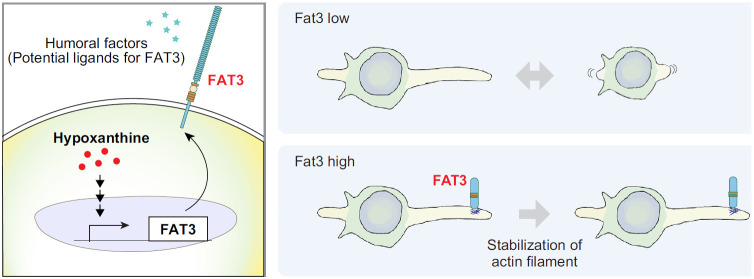
A model describing the functions of FAT3 in microglia. FAT3 expression is induced by hypoxanthine in microglia. FAT3 localized on the tip of the microglial process may stabilize actin reorganization in concert with a humoral factor.

In this study, we propose that the FAT atypical cadherin family protein, FAT3, is a novel mediator that controls the morphology of microglia cell line BV2. We also found that hypoxanthine induces FAT3 expression. Moreover, FAT3 in concert with a potential humoral factor suppresses the retraction of their processes. Thus, it is likely that the hypoxanthine-Fat3 axis is crucial for regulating the microglial morphologic changes (Fig. 5).

In the nervous systems, cadherin family proteins, including FAT-related protein, play an important role in regulating a variety of neuronal aspects, such as neuron-glia interaction, synaptogenesis, axon patterning, and dendritic arborization (Takeichi, 2007). So far, it has been shown that FAT3 and FAT1 are thought to be involved in actin rearrangement through interacting with ENA/VASP proteins (Moller et al., 2004; Tanoue and Takeichi, 2004; Krol et al., 2016). Interestingly, FAT3 subcellular localization is not uniform but limited in a specific site. For instance, FAT3 has been reported to be asymmetrically localized to the processes in the inner plexiform layer in retinal amacrine cell precursors. The opposite processes of amacrine cells are retracted, resulting in forming unipolar cells (Deans et al., 2011; Krol et al., 2016). In accordance with this research, we found that FAT3 in microglia is necessary for stabilizations of the processes in BV2 cells (Fig. 4*A–C*). Moreover, we observed that FAT3 subcellular localization does not tend to be diffusive at the peripheral region but concentrated locally. Therefore, we considered that asymmetric FAT3 anchors actin-related protein such as ENA/VASP and controls the actin rearrangement under the FAT3-expressed region. As FAT-related proteins contribute to homophilic or heterophilic cell adhesion (Nakayama et al., 2002; Ishiuchi et al., 2009), FAT3-interacting protein may have influences on the cell-cell contact or actin organization. So far, it has been reported that *Drosophila* Fat protein interacts with another cadherin family protein Dachsous (Ds), contributing to the regulation of planar cell polarity (Matakatsu and Blair, 2004). Intriguingly, the functions of both Fat and Ds are regulated by Four-jointed, which phosphorylates the Fat and Ds in the Golgi apparatus (Ishikawa et al., 2008). We found that expression level of FAM20C, which is a secretary kinase as well as Four-jointed, is also increased when BV2 cells are cultured under DMEM/F12 medium (Fig. 1*D*). Therefore, it may be possible that a high-nutrient condition could promote expressions of not only FAT3 but also FAM20C, leading to enhancing the interaction between FAT3 and target cadherin via FAT3 phosphorylation. Meanwhile, we found that the humoral factors secreted from BV2 cells have influences on their morphologic changes (Fig. 4*D–H*). Since proteomic analyses had revealed that FAT3 is associated with several humoral factors, such as XCL1 and PDGFB (Huttlin et al., 2017), the FAT3-dependent morphologic changes is regulated by not only cell-cell interaction but also other extracellular proteins.

Although several purinergic receptors exist in microglia, we consider that microglial morphology relies on intracellular hypoxanthine for the following reasons; First, P0 purinergic receptors do not recognize hypoxanthine (Bender et al., 2002). Second, treatment with PPADS, which is an antagonist for P2X receptors, had little effect on changing BV2 morphology. Third, increasing intracellular hypoxanthine induces morphologic changes of BV2. An important question is how hypoxanthine mediates FAT3 expression. It has been known that hypoxanthine mediates salvage pathways to synthesize nucleotides. Hypoxanthine-guanine phosphoribosyl transferase (HPRT1), which catalyzes hypoxanthine to inosine monophosphate (IMP) is one of the key enzymes that regulate the salvage pathway. Previously, HPRT1 expression level has been reported to be changed in microglia around P14 (Bennett et al., 2016). The proteomics analyses have revealed that HPRT1 interacts with several transcription factors, such as Jun and NF-κB (Li et al., 2015). Thus, it could be possible that hypoxanthine regulated HPRT1 cooperates with these transcription factors and upregulates FAT3 expression. Intriguingly, mutations in *Hprt1* gene cause Lesch–Nyhan syndrome (LNS) (Yang et al., 1984), which is a rare neurologic disorder exhibiting accumulation of uric acid caused by a defect in nucleic acid metabolisms. Because a decrease of uric acid accumulation does not cure the LNS symptoms, there is another possibility that aberrant microglial functions via abnormal hypoxanthine metabolism may cause neurologic disorders. The salvage pathway, rather than the *de novo* pathway, functions preferentially in the adulthood brain. On the other hand, it seems that the *de novo* pathway is activated dominantly under the postnatal stage, indicating that the switching from the *de novo* to the salvage pathways could occur after birth. Given that the upregulation of intracellular hypoxanthine concentration promotes HPRT1 activity, HPRT1 activation could be a key event that triggers the microglial transition under postnatal brains. Thus, it may be possible that loss of HPRT1 function causes the aberrant microglial transition, resulting in the onset of neuropsychiatric disorders, such as LNS.

BV2 cells are often used to be in place of primary microglia in an *in vitro* experiment because the gene expression patterns in BV2 cells are similar to the primary microglia under the reactive state (Henn et al., 2009). In addition, the analyses of BV2 cells have contributed to understanding the molecular mechanisms of intracellular and intercellular phenomena, such as caspase signaling dependent microglia activation (Burguillos et al., 2011), the microglia and glioblastoma communication (Shen et al., 2016), and microglial phagocytosis (Pluvinage et al., 2019). Moreover, BV2 cells are used to facilitate investigating the mechanisms of the neuronal disorder, such as Alzheimer’s disease, Huntington’s disease, amyotrophic lateral sclerosis, and stroke (Urushitani et al., 2006; Burguillos et al., 2015; Zhao et al., 2018; Joshi et al., 2019; Pluvinage et al., 2019), indicating that BV2 cells are a powerful tool to study the microglial functions. On the other hand, the shape of surveillant microglia *in vivo* is different from in BV2 cells.

FAT3 has been reported to be localized at the process in retinal amacrine cells and suppresses their retraction. Since FAT3 interacts with ENA/VASP protein, it is likely that FAT3 regulates a local actin reorganization in neurons. As well as amacrine cells, we found that treatment with hypoxanthine stabilizes their morphology of BV2 cells. Thus, we presume that one of FAT3 functions is to control actin rearrangement and stabilize processes locally. The interesting question is how subcellular localization of FAT3 is regulated. Since FAT3 is a cadherin family protein, the target proteins expressed in other cells could influence on subcellular localization of microglial FAT3. Although this is an interesting enigma, a detailed analysis will be a need in the future.

In conclusion, we found that FAT3 regulates BV2 morphology in coordination with a humoral factor derived from BV2 cells. The expression level of FAT3 in BV2 is regulated by intracellular hypoxanthine in microglia, demonstrating that the hypoxanthine-FAT3 axis is a novel pathway that links microglial morphologic changes to their traits. Since defects in *Fat3* gene are associated with several neuropsychiatric diseases, such as schizophrenia, autism, and ataxia (Yan et al., 2016; Baron et al., 2019; Capkova et al., 2019), our finding may provide a hint that links microglial dysregulations to the onset of these diseases.

## Synthesis

Reviewing Editor: Quentin Pittman, University of Calgary Decisions are customarily a result of the Reviewing Editor and the peer reviewers coming together and discussing their recommendations until a consensus is reached. When revisions are invited, a fact-based synthesis statement explaining their decision and outlining what is needed to prepare a revision will be listed below. The following reviewer(s) agreed to reveal their identity: NONE.

The reviewers and I agree that you appear to have evidence for a novel role for Fat3 in determining morphology in a mouse microglial cell line. The primary finding is that an increase in the intracellular amounts of hypoxanthine in a microglial cell line (BV2) leads to increased production and then localization of Fat3 protein, which is in turn leads to changes in BV2 morphology. Evidence implicating Fat3 is based on well-quantified and well-controlled changes in mRNA levels as well as siRNA knockdown. Additional strengths are that experiments were repeated multiple times and the paper’s conclusions are largely supported by the experiments. However, there are also some critical issues that need to be addressed, as well as numerous problems with the text and citations.

Revisions required (major):

1.The significance of this study is limited due to its reliance entirely on experiments performed with a transformed cell line. The process of immortalization, not to mention the genetic drift that occurs over multiple passage numbers, always introduces differences between the derived line and the original cell type from which it was derived. An attempt should be made to confirm the basic findings of this study with primary microglia. Thus in primary microglia you must provide evidence that hypoxanthine elevates Fat3 expression.

2.Relatedly, the types of morphological difference that this study highlight are often associated with different activation states of microglia. As they become more inflammatory, microglia in an intact CNS retract their processes and become more “amoeboid” or rounded. It is not clear what is meant by “polarization” or how “polarization” was measured throughout the paper. A great deal of the data rely on “relative percentage of polarized cells,” but there is no description of how this value was obtained. Was it through subjective evaluation by a human observer? And was this person aware of the treatment conditions? This makes it difficult to assess the assay, which is central to the study. The BV2 cells appear elongated, but I am not sure how this is an indication of “polarity”. Additionally, the cells do not appear at all like microglia, leaving it unclear whether the assay is measuring something physiologically relevant. It is incumbent upon the authors to discuss this phenomenon. It would also be nice to know if hypoxanthine can also suppress other markers of inflammatory activation, such as expression of NOS2 or various cytokines. Thus you should provide evidence that hypoxanthineand/or manipulation of Fat3 alters some index of activation as we are unwilling to accept morphological change as a meaningful endpoint even in the BV2 cell line.

3.The siRNA experiment must include a control transfection of non-complementary RNA.

4.This study describes generation of a new Fat3 antibody that is validated by showing loss of the signal in BV2 cells treated with siRNA against Fat3. It would be valuable to provide additional validation of the antibody, for instance on Western blot or in vivo. In particular, if it works for immunostaining, some information about the endogenous Fat3 protein distribution in microglia would be valuable.

5.There is one major inconsistency that absolutely requires clarification. The experiments show that extracellular hypoxanthine has no effect on microglia morphology but that intracellular hypoxanthine does. However, the very first finding is that addition of extracellular hypoxanthine (i.e. via DMEM/F12) induces Fat3 expression and alters microglia shape. Please explain how addition of extracellular hypoxanthine in the medium is able to induce intracellular hypoxanthine if this is not via entry into the cell (as blocked using a P2X purinergic receptor antagonist).

6.The identity of a possible extracellular cue that works in concert with Fat3 is interesting, but needs further substantiation and explanation. First, more information about the source of the conditioned medium is needed. Please explain how the BV2 cells were cultured, what density, for how long etc. Second, as shown in Fig. 4F, it seems that the cells were cultured for 24 hours first in DMEM/F12 and then moved either to fresh medium or conditioned medium for the next 6h (as indicated in the figure legend). If this is the case, the last paragraph (lines 279-280) should say “Therefore, hypoxanthine-induced fat3 expression is not sufficient for altering BV2 morphology further, implying that BV2 morphogenesis is mediated by extracellular factors as well.” This is more accurate as all of the cells were induced for 24h in DMEM/F12 first.

7.Statistics: Student’s t-test is not appropriate for data sets that have more than two groups. ANOVA should be paired with an appropriate post-hoc test to determine p-values.

Revisions suggested ( Minor, but should be addressed):

1.Although most results are appropriately quantified, it is often hard to know how to interpret individual findings given unexplained variation across the study. For instance, the basal percentage of elongated cells (i.e. in DMEM) varies quite a lot (ca. 10% in Fig. 1B, ca. 17% in 1J, ca. 8% in 2F, ca. 6% in 3C and ca. 7% in 3H. That is a delta of 9%). The figure legends give the impression that each data point of a particular graph corresponds to an independent experiment and there they are quite similar, so this makes it hard to understand what the source of variation might be. It is also unclear what the “n” corresponds to (multiple experiments on the same day, different days etc). The degree of the effect also varies significantly. In Fig. 1B, DMEM/F12 induces about 15% of the cells to polarize and then in Fig. 1J, it looks like ∼25% (there are other examples, too; I have not listed them all). For hypoxanthine, ∼15% of the cells are polarized in Fig. 2F and 3C but then the DY and ALP treatments induce 40% and 25% respectively. I know these are different treatments, but it warrants explanation nonetheless. Indeed, unexpectedly high values are also reported in Fig. 4F, where hypoxanthine (with fresh media) induces ∼30% of the cells to polarize. There is a similar problem in Fig. 2B/2C, where there is an order of magnitude difference in the qRT-PCR values. This does not make sense if all the data points either within a graph or across graphs correspond to independent experiments. This kind of variability is disconcerting and undermines the strength of the findings.

2.The identity of a possible extracellular cue that works in concert with Fat3 is interesting, but needs further substantiation and explanation. First, more information about the source of the conditioned medium is needed. Please explain how the BV2 cells were cultured, what density, for how long etc. Second, as shown in Fig. 4F, it seems that the cells were cultured for 24 hours first in DMEM/F12 and then moved either to fresh medium or conditioned medium for the next 6h (as indicated in the figure legend). If this is the case, the last paragraph (lines 279-280) should say “Therefore, hypoxanthine-induced fat3 expression is not sufficient for altering BV2 morphology further, implying that BV2 morphogenesis is mediated by extracellular factors as well.” This is more accurate as all of the cells were induced for 24h in DMEM/F12 first.

3.Since one of the major messages is that the induction of Fat3 leads to the observed change in BV2 cell shape, it would be interesting to see if overexpression of Fat3 is sufficient. I understand this could be a challenging experiment, due to the large size of Fat3, but it would strengthen the findings if it is possible. For instance, it would also be possible to test if the conditioned medium has an effect only in cells with Fat3 overexpression. In this regard, it has been noted that primary microglia grown in an antioxidant-rich serum-free medium such as Neurobasal/B27 have a more complex, process-bearing morphology consistent with “resting” cells in vivo. If the whimsy struck them, the authors might consider testing the role of FAT3 in this morphological change as well. This should not be considered mandatory, however.

4.The figure legends are poorly written. It is difficult to determine what the data in the figures represent from reading the legends. A figure legend should contain basic information about what sort of experiment was conducted, how the data were collected, and details of concentration and time of treatments. The legend for Figure 1 makes mention of “the postnatal stage,” but BV2 cells are not born. From what experimental model are these data derived? And is the graph in Panel D “modified” from a graph in Brain RNA-Seq database, or was it produced outright from numerical data found there? And what is the relationship of the data to Tmem119? Are these Fat3 mRNA levels in Tmem119-positive and -negative cells? How were the Tmem119-negative cells related to “microglia”?

5.There are frequent difficulties with sentence structure, verb tense and language and you are urged to have the manuscript edited by someone with a greater command of the English language. Example of awkward sentence structure include: “To do this, we first checked the efficiency of FAT3 siRNA. In consequence, treatment with siRNA significantly reduced expression levels of both mRNA and protein (Fig. 1G and 1H).” It is not a consequence of checking the efficiency of the siRNA that the treatment reduced expression (unless it is true that a tree falls silently in the forest if no one is there to hear it).

"BV2 cells have been thought to be the alternative cells as a replacement for primary microglia because the gene expression patterns in BV2 cells are similar to the primary microglia under the reactive state (Henn et al., 2009).” Use of the phrase “the alternative cells” implies that there is no other cell line used in this way. There is no consensus among researchers that BV2 are preferable to CHME3, N9, HAPI, or several other lines used to model microglia.

"Moreover, using BV2 cells has revealed the underlying mechanisms of neuronal disorder, such as Alzheimer’s disease, Huntington’s disease, amyotrophic lateral sclerosis and stroke....” As someone who has watched the scientific enterprise hammer away at these disorders unsuccessfully for 35 years, I find it quite surprising that someone would claim that we know the underlying mechanisms of these conditions.

6.Several phrases are misused that could lead to confusion among readers (such as “in consequence", line 203 and elsewhere).

Line 151: Cells in culture should not be referred to as “tissue.”

Line 210-211: The text should be rewritten to more accurately reflect the experiment and finding: “treatment with siRNA significantly reduced DMEM/F12-induced expression levels of both mRNA and protein.” Additionally, the levels of protein were not actually measured, and this should be made clear.

Line 230: It is unclear what is meant by “local.”

7.Not all relevant citations are included - see line 298, line 307, line 309 for example. Please make sure that all statements are properly referenced.

8.Figure 5 is not really a “model.” It adds nothing to the readers’ understanding of the study.
